# Critical interactions between opioid and cannabinoid receptors during tolerance and physical dependence development to opioids in the murine gastrointestinal tract: proof of concept

**DOI:** 10.1007/s43440-021-00291-7

**Published:** 2021-06-16

**Authors:** Agata Szymaszkiewicz, Mikołaj Świerczyński, Marcin Talar, Prabhakar Reddy Polepally, Jordan K. Zjawiony, Jakub Fichna, Marta Zielińska

**Affiliations:** 1 Department of Biochemistry, Faculty of Medicine, Medical University of Lodz, Mazowiecka 6/8, 92-215 Lodz, Poland; 2 Department of Biomolecular Sciences and Research Institute of Pharmaceutical Sciences, University of Mississippi, Oxford, MS USA

**Keywords:** Co-activation, Cross-talk, Opioids, Physical dependence, PR-38, Tolerance

## Abstract

**Introduction:**

Tolerance (TOL) and physical dependence (PD) constitute important limitations of opioid therapy. The aim of our study was to validate research tools to investigate TOL and PD and to characterize the interactions between opioid (OR) and cannabinoid (CB) receptors in these processes in the GI tract.

**Methods:**

TOL was assessed through the comparison of morphine ability to inhibit electrically evoked smooth muscles contractility in the mouse ileum that was previously incubated with/without morphine for 1 h. To evaluate the PD, the ileum was incubated with morphine for 10 min, then challenged with naloxone to induce withdrawal response (WR). The OR/CB interactions were evaluated using mixed agonist (PR-38) and AM-251 (CB1 antagonist).

**Results:**

The inhibitory effect of morphine on ileal contractions was weaker in tissue incubated with this opioid than in tissue incubated without opioid. The opposite was noted for PR-38. In tissues exposed to morphine, but not to PR-38, naloxone induced a WR. The blockage of CB1 receptors with AM-251 before the addition of PR-38 resulted in a naloxone-induced WR.

**Conclusion:**

The co-activation of OR and CB reduced development of TOL and PD to opioids in the mouse GI tract and mixed OR/CB agonists are promising alternative to currently used opioid drugs.

## Introduction

For centuries, opioids have been used as effective therapeutics in pain management. These agents mediate pharmacological effect through the activation of opioid receptors (ORs: MOP, KOP, DOP receptors) [1]. Development of tolerance and physical dependence to the analgesic effect of opioids is one the most important side effects during chronic therapy with these drugs. The development of tolerance evokes a gradual decrease of opioid activity. The increase of drug dose is required to overcome tolerance and induce adequate pain relief. Together with the increment of a drug dose, a risk of other adverse effects increases, i.e. physical dependence. Physical dependence is a condition, in which abrupt or gradual drug withdrawal or administration of antagonist (in case of opioids—naloxone) causes unpleasant physical symptoms, such as: weight loss, diarrhea, jumping, teeth chattering. These symptoms disappear after the administration of drug or over time [2, 3].

Noteworthy, tolerance and physical dependence involve the whole organism including the GI tract [4]. It was found that patients chronically treated with morphine develop tolerance to its analgesic effect, but not to constipatory action of this opioid [3]. Particular parts of the GI tract differ significantly in their response to opioids, for example tolerance occurs in the ileum, but not in the colon [4–8].

Experimentally, tolerance and physical dependence can be induced either by in vivo exposure to opioids or in vitro incubation of the isolated tissue from naive animals with OR agonists. The measure of tolerance development is a decrease of drug activity, while the sign of physical dependence is the presence of so-called ‘withdrawal response’ (WR) following the administration of antagonist (commonly naloxone). Naloxone evokes a sudden release of excitatory neurotransmitters (acetylcholine and substance P) from neurons, what results in a rapid increase in the contractility of the smooth muscles (counted as WR) [9–11]. This WR can be induced by naloxone even in tissue shortly exposed to opioid (few minutes) [12].

One of the strategy of minimization of the adverse effects related to opioid therapy includes the simultaneous activation of ORs and cannabinoid (CB1, CB2) receptors. This approach is promising due to the cumulative or synergistic effect in vivo and also according to lower potency to induce tolerance and physical dependence related to the analgesic activity of opioids.

In our study we validated two novel in vitro methods that can be applied for fast screening of the following pharmacological properties of future drugs: the development of tolerance and physical dependence in the longitudinal preparations of the mouse ileum. Moreover, as little is known about the influence of the activation of CB receptors in the development of tolerance and physical dependence to opioids in the GI tract, we aimed to evaluate the effect of co-activation of OR and CB receptors (using a mixed OR/CB1 agonist: PR-38) on these phenomena in the mouse GI tract [13, 14].

## Materials and methods

### Animals

In this study, male Balb/C mice weighing from 22 to 26 g were used (purchased from The Institute of Occupational Medicine, Lodz, Poland). Animals were maintained at the constant temperature (22–23 ℃) under 12-h light/dark cycle. Mice were housed in sawdust-lined plastic transparent cages with a free access to laboratory chow and tap water. All of the experiments in this study were performed in accordance with respective national guidelines.

### Tissue isolation

Mice were sacrificed by cervical dislocation. Subsequently, the ileum was gently removed and washed with Tyrode solution (NaCl 115.0 mM, KCl 8.0 mM, KH_2_PO_4_ 2.0 mM, NaHCO_3_ 25.0 mM, MgCl_2_ 2.4 mM, CaCl_2_ 1.3 mM, glucose 10.0 mM). Full-thickness fragments of the ileum (0.5 cm) were kept in Tyrode solution. One end of each ileal fragment was attached using a silk thread to the bottom of the individual organ bath, another end to a FT03 force displacement transducer (Grass Technologies, West Warwick, RI, USA).

Each organ bath contained 25 ml of Tyrode solution oxygenated with 95% O_2_ and 5% CO_2_ at constant temperature (37 °C). The changes in tension were amplified by a P11T amplifier (Grass Technologies, West Warwick, RI, USA) and recorded using the POLYVIEW software (Polybytes Inc., Cedar Rapids, IA, USA).

### Tolerance development

To induce tolerance to opioid, the mouse ileal preparations were incubated in Tyrode solution containing morphine (10^–6^ M) for 60 min. After this incubation, there was a wash out (Tyrode buffer containing opioid was replaced with fresh Tyrode solution) and tissue was stimulated with electrical field (S88X stimulator, Grass Technologies, 8 Hz, 60 V, pulse duration 0.5 ms, train duration 10 s), delivered through electrodes placed around the tissue. After the 25 min equilibration period, the mean amplitude of three twitch contractions was measured and treated as an internal control. To assess whether tolerance has developed in the ileum, morphine was added cumulatively at increasing concentrations into the organ baths (10^–11^ to 10^–6^ M, 8 min for each concentration). The amplitude of the smooth muscle contractions was measured after the addition of each dose of opioid and referred as the percentage of the internal control.

**Fig. 1:**
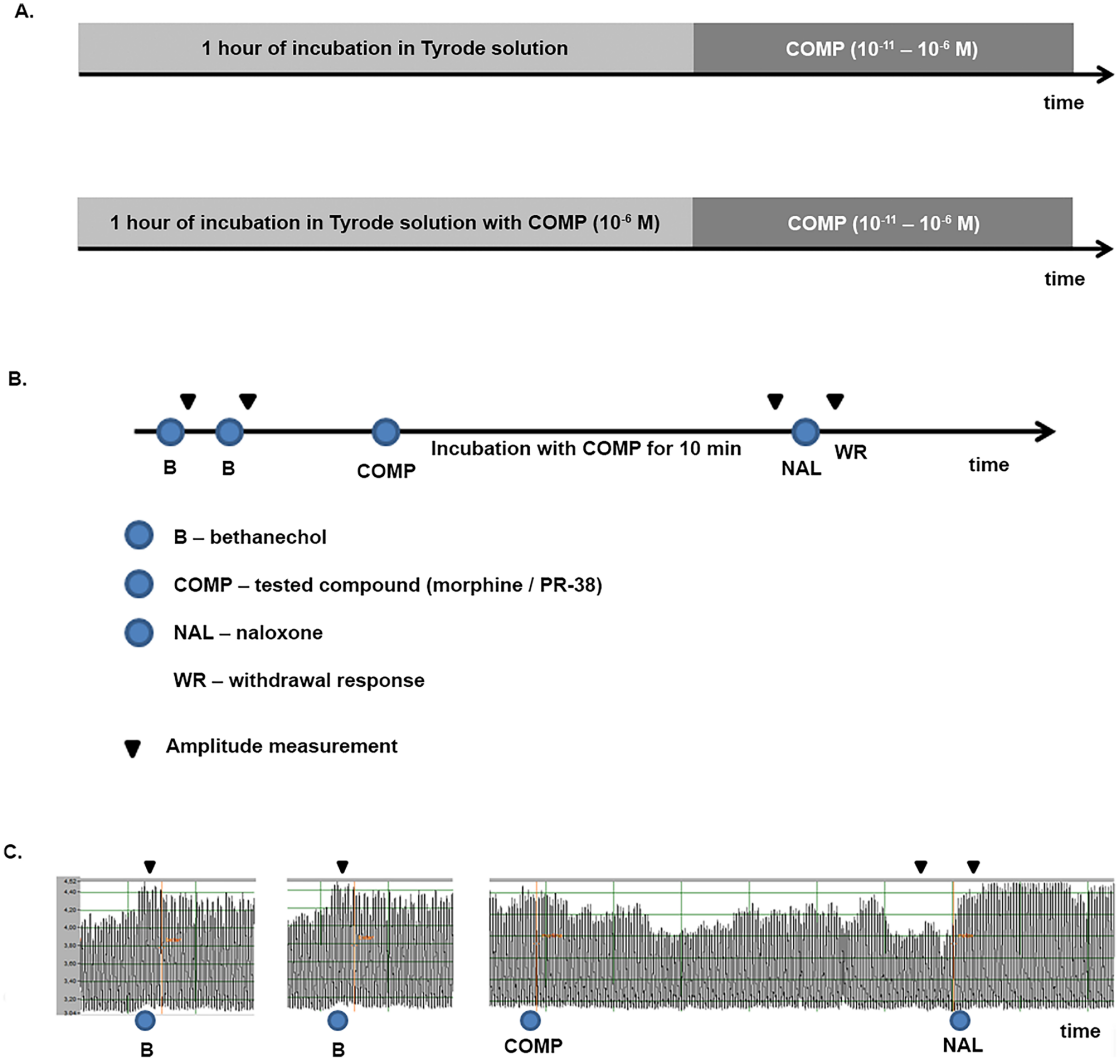
Procedures in the assessment of tolerance (**A**) and physical dependence (**B**). The recordings from the experiment with morphine, which present the course of the physical dependence set up (**C**)

The inhibitory effect of morphine (10^–11^ to 10^–6^ M) on electrical field stimulation (EFS)-smooth muscle contractility was compared between the tissues that were incubated with morphine for 60 min (tissue that became tolerant to morphine) to ‘naïve’ preparations that were incubated with Tyrode solution without opioid. The sequence of procedures of the experiment with morphine is presented on the Fig. 1 (Fig. 1A).

The same setup was used to evaluate the potency of PR-38 to develop tolerance in the ileum. In that case, PR-38 was used instead of morphine (at the same concentration as morphine).

### Naloxone-induced withdrawal response

At the beginning of the set up, the ileal tissues were challenged with bethanechol (10^–6^ M, added twice), which evoked strong contractions of longitudinal smooth muscles referred as an internal control (mean of two measures). Then, morphine (10^–6^ M) was added into the organ bath. After 10 min of incubation with opioid, tissues were challenged with OR antagonist, naloxone (10^–6^ M). An observed increment of the smooth muscle contractility immediately after exposure to naloxone was considered as withdrawal response (WR), a measure of physical dependence development (Fig. 1B, C). Although, each ileal preparation was used twice, in results section we present only the first experiment as the results were comparable. Morphine was used to validate this method: we adjusted the duration of exposure to opioid (3–15 min), the moment of amplitude measurement after the naloxone addition to the organ bath. Naloxone at concentration of 10^–6^ M and 10 min of incubation was selected as optimal conditions according to the repeatability and reproduction of results.

To evaluate whether CB receptors are involved in the occurrence of naloxone-induced WR we performed experiments with WIN 55,212-2, a potent CB receptors agonist.

After validation of this method we assessed the potency of mixed OR/CB1 agonist, PR-38, to induce physical dependence in the mouse ileum. Noteworthy, to determine the involvement of CB receptors component in the development of physical dependence, the activity of CB1 receptors was blocked with AM-251 (10^–7^ M). AM-251 was added 10 min before the PR-38 into the organ bath.

### Drugs

All components of Tyrode solution were purchased from Sigma-Aldrich (Poznan, Poland). Naloxone hydrochloride, WIN 55,212-2 and AM-251 hydrochloride were purchased from Tocris Bioscience (Ellisville, MO, USA). Morphine sulfate was obtained from Polfa (Warsaw, Poland). 2-*O*-cinnamoyl-salvinorin B (PR-38) was synthesized in the Department of Biomolecular Sciences and Research Institute of Pharmaceutical Sciences (University of Mississippi, MS, USA) [15].

In the in vitro experiments, all drugs were dissolved in dimethyl sulfoxide (DMSO). Importantly, DMSO alone had no effects on the observed parameters.

### Statistics

In the in vitro experiments, *n* indicated the number of individual tissues from ≥ 3 different animals. Statistical analyses were performed using PRISM 9.0 (GraphPad Software Inc., La Jolla, CA) and Statistica 13 software (StatSoft, Tulsa, OK, USA). The Shapiro–Wilk test was used to confirm the Gaussian distributions of raw data, as well as the distributions of differences between pairs in the case of paired comparisons. Dose–response curves in tolerance development experiments were fitted by non-linear regression (the log(inhibitor) vs. response equation, and the extra-sum-of-squares *F* test was used to compare the groups of morphine incubated with Tyrode containing morphine vs. morphine incubated with Tyrode alone and PR-38 incubated with Tyrode containing PR-38 vs. PR-38 incubated with Tyrode alone.

The significance of the effects of the smooth muscle contractions, interpreted as withdrawal response on upon utilizing MOR/WIN and NAL or PR-38 (alone and in the presence of AM-251) and NAL was tested by the non-parametric Wilcoxon signed rank test. *p* values < 0.05 were considered statistically significant.

## Results

We used morphine to validate an in vitro method to assess development of tolerance in the mouse ileum. We observed that morphine (10^–11^ M–10^–6^ M) inhibited EFS-induced smooth muscles contractions in a concentration-dependent manner.

**Fig. 2:**
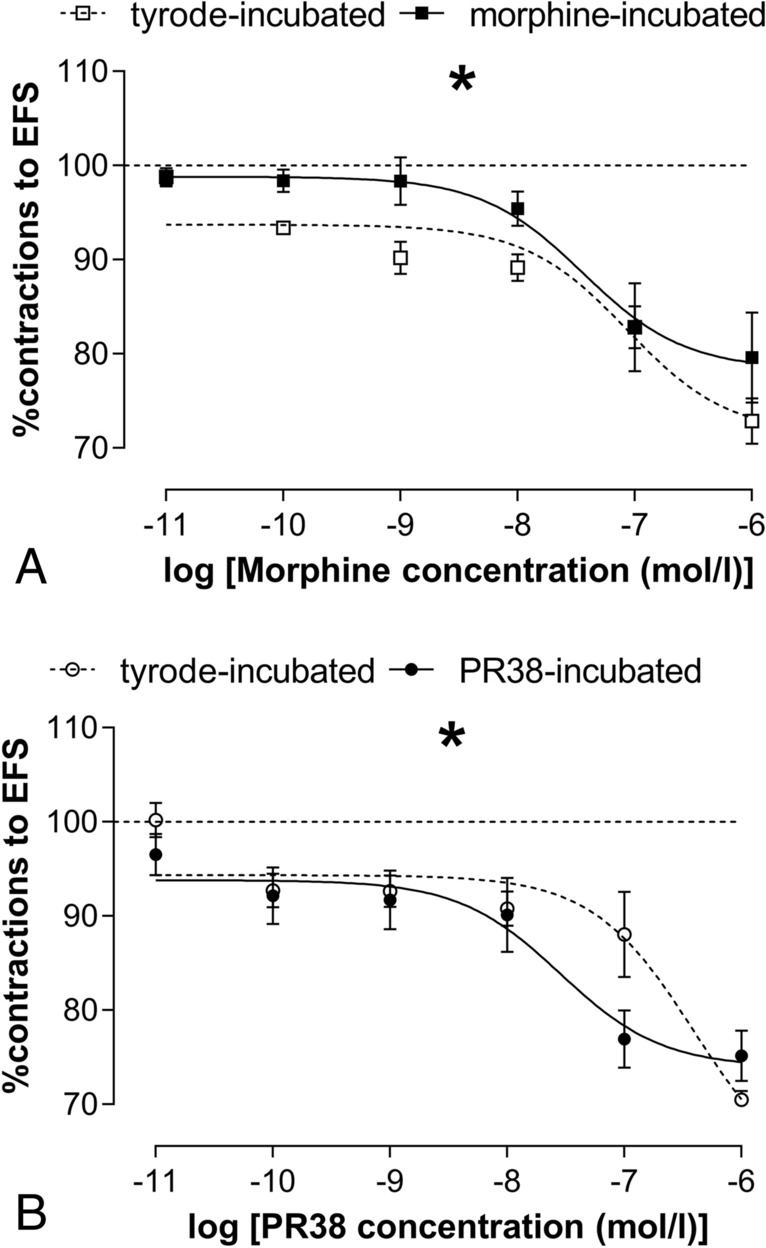
**A** The assessment of the development of tolerance after 1 h incubation in Krebs solution containing morphine or without opioid. Dose-dependent effects of morphine concentration on % concentrations to EFS in morphine-incubated and Tyrode-incubated ileum. Data is presented as a mean ± SEM; *n* = 6 while n indicated the number of individual tissues from ≥ 3 different animals for morphine-incubated ileum (solid line, black squares) and Tyrode-incubated ileum (dashed line, white squares). **B** Tolerance development in the ileum incubated with PR-38 or vehicle for 1 h. Dose-dependent effects of PR-38 concentration on % concentrations to EFS in PR-38-incubated and Tyrode-incubated ileum. Data are presented as a mean ± SEM; *n* = 6 while n indicated the number of individual tissues from ≥ 3 different animals for PR-38-incubated ileum (solid line, black circles) and Tyrode-incubated ileum (dashed line, white circles). The curves (**A**, **B**) were fitted to the three-parameter dose–response non-linear regression curve. *p* = 0.0331 for Fig. 2A and *p* = 0.0445 for Fig. 2B, based on the extra-sum-of-squares *F* tests

This inhibitory effect of morphine was significantly weaker in tissue preparations that were previously incubated with morphine for 60 min in comparison to the ileal segments that were incubated without opioid (Fig. 2A). This difference is a result of the development of tolerance following 60 min incubation with OR agonist.

In further studies we evaluated whether a mixed OR/CB1 agonist (PR-38) differs from morphine in its potency of tolerance development in the mouse ileum. We observed that the effect evoked by PR-38 added into the organ bath at increasing concentrations (10^–11^–10^–6^ M) was opposite to that obtained in experiments with morphine: the incubation with PR-38 did not lead to the tolerance development. Interestingly, we found that tissue incubated with PR-38 was even more sensitive to further action of agonist that tissue incubated without PR-38. Noteworthy, at lower concentrations (10^–11^–10^–9^ M) the inhibitory effect of PR-38 was equal in the tissue incubated with and without PR-38 for 60 min (lack of tolerance), while at higher concentrations (10^–8^–10^–6^ M) the concentration–response curve for PR-38 was shifted to the left in the tissue incubated previously with this agonist (the increment of the sensitivity to the inhibitory action of PR-38) (Fig. 2B).

**Fig. 3:**
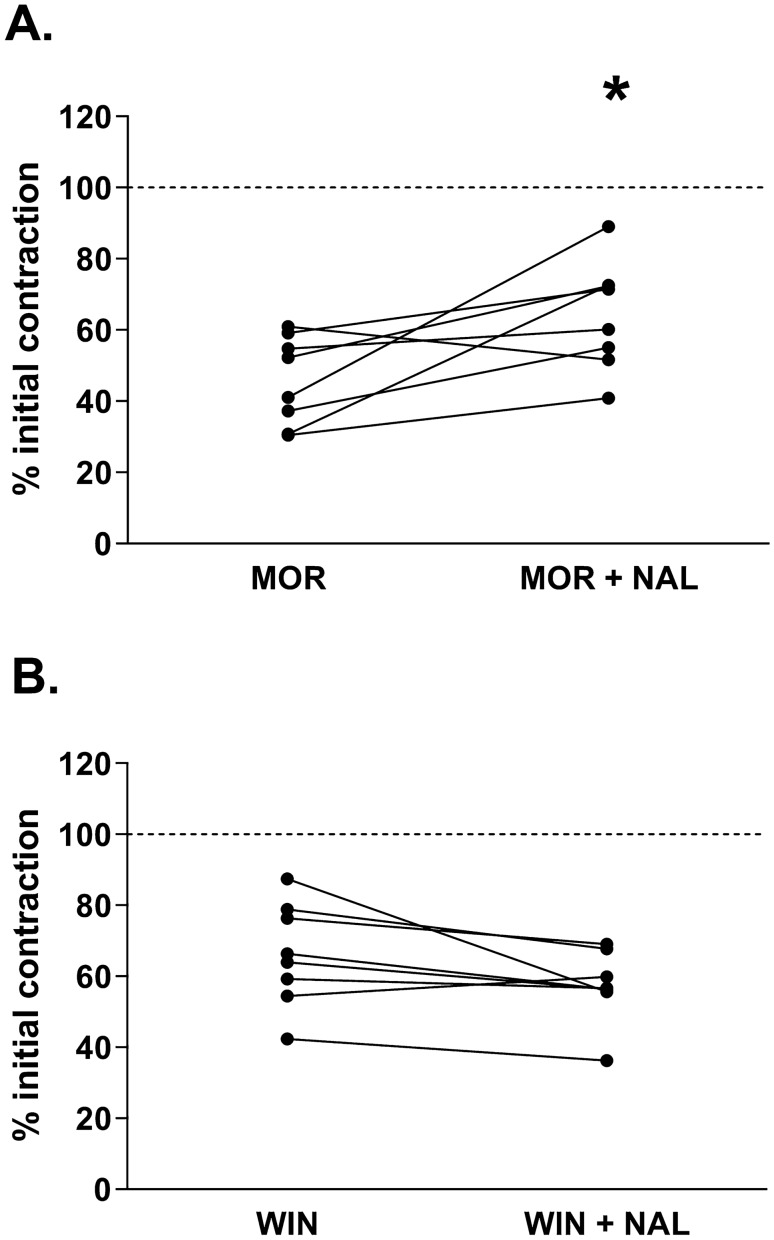
The presence of naloxone-induced withdrawal response (expressed as an increase in the amplitude of smooth muscles contractility) in the ileum incubated for 10 min with morphine (MOR) (**A**) or WIN 55,212–2 (WIN) (**B**). The graphs present the amplitude contractions referred as a percentage of bethanechol induced contractions (internal control—dashed line). The amplitude of contractions in morphine-dependent tissue was measured right before the addition of naloxone and then immediately after naloxone administration into the organ bath. Data presented as ladder plots. Significance estimated with the use of Wilcoxon’s signed rank test. In experiments with morphine *p* = 0.0234 (*z* = 2.2404, *N1* = 8, *N2* = 8) as compared to morphine prior the addition of naloxone. In experiments with WIN 55,212-2, the results were not statistically significant with *p* = 0.0645 (*z* = 1.8857, *N1* = 10, *N2* = 10) as compared to WIN 55,212-2 prior the addition of naloxone

An in vitro method that enables to evaluate development of physical dependence was validated using morphine. We observed that in the mouse ileal segment incubated with morphine (at 10^–6^ M) for 10 min, subsequent exposition to naloxone resulted in a significant and rapid increase in the amplitude of the smooth muscle contractions, interpreted as WR (Fig. 3A). The presence of WR is a sign that physical dependence has developed after the 10 min incubation with morphine. In the experiments with CB receptors agonist, WIN 55,212-2, there was no increment of the amplitude of the smooth muscle contractions after the addition of naloxone (Fig. 3B). These results indicate that naloxone-induced WR occurs only in case of activation of OR, not CB receptors.

**Fig. 4:**
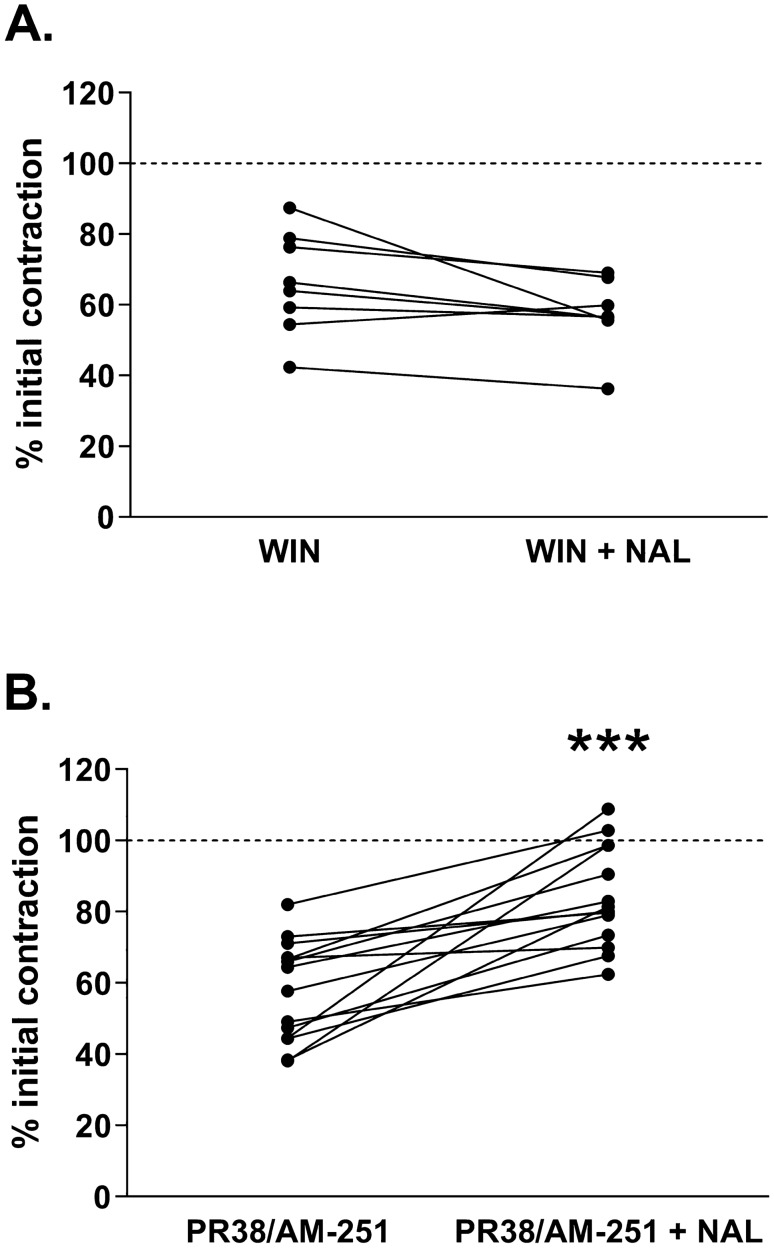
The presence of naloxone-induced withdrawal response (expressed as an increase in the amplitude of smooth muscles contractility) in the ileum incubated for 10 min with PR-38 (**A**). Panel B shows the presence of WR in tissue pretreated with AM-251 prior the addition of PR-38 (**B**). The graphs show the amplitude contractions referred as a percentage of bethanechol induced contractions (internal control—dashed line). The amplitude of contractions in morphine-dependent tissue was measured right before the addition of naloxone and then immediately after naloxone administration into the organ bath. Data presented as ladder plots. Significance estimated with the use of Wilcoxon’s signed rank test. In experiments with PR-38 alone the results were not statistically significant with *p* = 0.5186 (*z* = 0.7060, *N1* = 12, *N2* = 12) as compared to PR-38 prior the addition of naloxone. In experiment with PR-38 + AM-251, *p* = 0.0001 (*z* = 3.2958, *N1* = 14, *N2* = 14) as compared to PR-38 + AM-251 prior the addition of naloxone

In experiments with mixed OR/CB1 agonist (PR-38), we assessed that in the tissue incubated previously with PR-38, the exposition to naloxone did not induce the WR (Fig. 4A). To evaluate the involvement of CB receptor component of PR-38 in the development of physical dependence, we blocked CB activity with antagonist, AM-251 (Fig. 4B). Noteworthy, in the ileal preparations exposed to CB antagonist prior to 10 min incubation with PR-38, the addition of naloxone evoked the WR. It indicated that the simultaneous activation of OR and CB1 receptors with PR-38 abolished the development of physical dependence to opioids in the GI tract. Concurrently, a blockage of CB1 receptor related signaling of PR-38 induced development of physical dependence.

## Discussion

Over the years, researchers investigated the possible options of reduction of the risk of tolerance and physical dependence related to the chronic application of opioids. Interestingly, development of tolerance to the analgesic action of morphine (attributed to the central nervous system, CNS) was accompanied by the tolerance to the inhibitory effect of this drug in the GI tract. However, tolerance occurred in the upper GI tract, but not in the colon [5]. In this study, we validated two in vitro methods that can be applied in the fast screening of drug ability to induce tolerance and physical dependence in the GI tract. Furthermore, as the results of animal studies showed that there are numerous factors that affect development of tolerance and physical dependence to the analgesic effect of opioids (i.e. activation of CB receptors), we evidenced that simultaneous activation of OR and CB receptors with PR-38, mixed agonist, abolished the development of tolerance and physical dependence in the mouse ileum.

Tolerance to opioids could be evoked either in vivo (repeated administration of drug) or in vitro (incubation of tissue with opioid). Ross et al. [5] reported that longitudinal preparations of the ileum from morphine-tolerant mice (animals with implanted pellet containing 75 mg of morphine) became resistant to stimulation with morphine in vitro (in organ baths): the inhibitory action of morphine on EFS-stimulated contractility in the ileum isolated from morphine pelleted mice was significantly weaker than that observed in the intestine from placebo-implanted or non-implanted mice. In earlier studies on the ileum of guinea pigs (GP), it was shown that the incubation or repeated exposure to morphine in vitro led to the development of tolerance to the effect of morphine on neurogenic contractions in the longitudinal muscles to the same extent as in vivo treatment with opioid [4, 6]. As both, in vivo and in vitro exposure to opioid could induce tolerance, in our study we adjusted an in vitro method of the assessment of tolerance development in the murine GI tract. Our results were in agreement with Rezvani et al. [6], as they found that 60 min incubation of GP ileum preparation with an OR agonist was enough to evoke acute tolerance. We assessed that 60 min time period of incubation with opioid led to the development of tolerance in the mouse ileum, as the inhibitory action of morphine on EFS-stimulated contractions was diminished in the preparations pre-incubated with Tyrode solution containing morphine nor Tyrode solution alone for the same time period.

Taking into consideration the beneficial effect of co-activation of OR and CB1 receptors (i.e. synergistic analgesic activity), we evaluated whether a cannabinoid component of mixed OR/CB ligand (PR-38, derivative of salvinorin A, SA) affected the tolerance forming potency of this compound. In our previous studies we found that SA activated KOP, CB1, CB2 receptors in the GI tract and thus attenuated colitis, modulated neurogenic ion transport in the colon and inhibited the GI transit [13]. However, SA could not be applied in clinics due to the psychoactive activity in the CNS. PR-38 shared some features with SA (agonistic activity at MOP, KOP and CB1 receptors), but it was deprived of the action in the CNS [14]. Using a newly validated method we assessed the potency of PR-38 to develop tolerance in the GI tract. We observed that inhibitory action of PR-38 in tissue incubated for 60 min in Tyrode solution containing PR-38 was not weaker than the effect evoked in the preparation incubated with Tyrode solution alone. In fact, we observed that at higher concentrations of this compound, incubation with PR-38 increased the sensitivity to further activation with the inhibitory action of this drug. We determined that simultaneous activation of OR and CB receptors with PR-38 attenuated the tolerance development in the mouse GI tract, what was in agreement with numerous reports on the impact of CB receptors activation on the analgesic effect of opioid. For example, according to Cichewicz et al. [16], an oral co-treatment with morphine (p.o. twice daily for 7 days: 200 mg/kg for 2 days; 300 mg/kg for latter days) and CB1 agonist, Δ9-tetrahydrocannabinol (Δ9-THC, 20 mg/kg, 15 min prior to morphine, twice daily) did not induce development of tolerance to antinociceptive effect of morphine. Interestingly, Basilico et al. [17] in the study on GP ileal myenteric plexus-longitudinal muscle preparations, reported the opposite on the role of interactions between OR and CB receptors in the tolerance development. In contrast to our study, they used two separate agonists: morphine (as OR agonist) and WIN 55,212–2 (as CB receptors agonist). First, it was assessed that after 5 h time period of incubation either with MOP agonist or CB receptor agonist, the tissues became less sensitive to the further inhibitory action of respective ligands, what indicated a development of tolerance in both cases. Second, it was reported by that 5 h incubation with WIN 55,212–2 (5 × 10^−8^ M) decreased twofold maximum inhibitory effect of morphine when compared to the tissue incubated without CB agonist. The same results were obtained in experiments on the tissue that became tolerant to morphine (5 h incubation with opioid, 10^−7^ M) and then acutely exposed to WIN 55,212-2 (8 × 10^−7^ M). It was determined that co-activation of OR and CB receptors augmented the tolerance development to opioid in the GI tract of GP.

The development of tolerance is usually accompanied by the occurrence of physical dependence. Thus, we attempted to adjust in vitro WR method that would enable to evaluate the potency of opioids to develop physical dependence in a fast and simple way. In experiments with morphine we measured that addition of naloxone into the organ bath induced a significant increase in the amplitude of the intestinal contractions, which we interpreted as a sign of acute physical dependence–withdrawal response (WR). In the past, Collier et al. [4] adjusted the method based on the assessment of WR in the GP ileal preparations. They used numerous set ups which varied in the concentration of opioid (normorphine 0.01–1 pM), time of incubation (2–24 h) and the temperature of incubation (5, 22 or 37 ℃). It was observed that in the ileum incubated with normorphine (2–24 h), the exposition to naloxone (0.03 pM) at 22 ℃ produced significant WR, while there was no WR response in the control tissue that was incubated without OR agonist. The WR was also induced in the tissue incubated with opioid after washout and exchange of the incubation buffer. Collier et al. [4] concluded that a single, short-term exposition to opioid was not enough to induce physical dependence, but Valeri et al. [12] found that even short (few minutes) exposition to opioid evoked physical dependence. A significant naloxone-induced WR was noted in the GP ileum incubated with morphine (10^–7^ M) for 5 min, what was similar to the results obtained in our study in which the exposition to morphine lasted 10 min.

To evaluate how the co-activation of OR and CB receptors with PR-38 affected the development of physical dependence, we examined the presence of naloxone-induced WR in the tissue incubated with this mixed agonist. The only one report on the involvement of CB receptors in the opioid-related physical dependence in the GI tract was obtained by Basilico et al. [17]. It was observed, that in GP ileal myenteric plexus preparations that became tolerant to morphine (5 h incubation, 10^−7^ M) the exposition to naloxone (10^−5^ M) resulted in strong WR. However, in the tissue samples that rendered tolerant to opioid, the addition of WIN 55,212-2 (5 × 10^−8^ M) 15 min before challenging with naloxone significantly abolished the WR. Similarly, in our study there was no naloxone-induced WR in the tissue incubated with PR-38. Both, our results and the experiments performed by Basilico et al., indicated that simultaneous activation of OR and CB receptors diminished the development of physical dependence. Noteworthy, we determined the role of cannabinoid component of PR-38 in the physical dependence development in the setups with CB antagonist, AM-251. In particular, strong WR was noted after the addition of naloxone in the tissue that was exposed to AM-251 prior to the incubation with PR-38. Therefore, we confirmed that the simultaneous activation of OR and CB receptors with mixed agonist resulted in reduction of physical dependence.

CB and OR exhibit several common features, such as: their neuroanatomical distribution, functional over-lapping (pain, reward process, anxiety, immune, or the action in the GI system), cellular co-localization and similarities in signal transduction. There are multiple possible mechanisms of cross-talk between MOP and CB receptors, as they both belong to the G protein—coupled receptors family and transduce signals via activation of Gi/o proteins, what is followed by inhibition of the adenylyl cyclase activity, activation of Ca2 + channel, neurotransmitter release and stimulation of the MAP kinase cascade [18, 19]. The interactions between OR and CB receptors could be a consequence of the ability of OR and CB receptors to form functional heterodimers [20]. Moreover, there is a possible contribution of allosteric modulation: agonist-occupied CB receptors act as an allosteric modulator of the partner opioid receptor and conversely [21]. For instance, it was reported that cannabidiol is a negative allosteric modulator of MOP: cannabidiol abolished the inhibitory action of DAMGO in the electrically induced twitch response test in the mouse vas deferens [22]. Interestingly, SA (parent compound to PR-38), besides being partial agonist of MOP, acts as a negative allosteric modulator of MOP [23]. Allosteric modulation of OR through activation of CB1 can potentially lead to the decreased potency of development of tolerance and physical dependence [21]. The beneficial effect of PR-38 on the tolerance and physical dependence development could result from the allosteric modulation of opioid receptors, but it needs to be determined if PR-38 is an allosteric modulator of OR in further research.

## Conclusion

In our study, we validated new pharmacological tools that allow to assess the development of tolerance and physical dependence in the mouse GI tract in vitro in a fast, inexpensive and simple way. Finally, we observed that co-activation of OR and CB receptors with PR-38 diminished the occurrence of tolerance and physical dependence in the mouse GI tract in vitro, further in vivo studies are warranted.

## Abbreviations

CB: Cannabinoid
CNS: Central nervous system
DOP: δ- Opioid receptor
EFS: Electrical field stimulation
GI: Gastrointestinal
GPI: Guinea pig ileum
KOP: ĸ- Opioid receptor
MOP: µ-Opioid receptor
OR: Opioid receptor
WR: Withdrawal response

## References

[1] Sobczak M, Sałaga M, Storr MA, Fichna J (2014). Physiology, signaling, and pharmacology of opioid receptors and their ligands in the gastrointestinal tract: current concepts and future perspectives. J Gastroenterol.

[2] Bihel F (2016). Opioid adjuvant strategy: improving opioid effectiveness. Future Med Chem.

[3] Ling GSF, Paul D, Simantov R, Pasternak GW (1989). Differential development of acute tolerance to analgesia, respiratory depression, gastrointestinal transit and hormone release in a morphine infusion model. Life Sci.

[4] Collier HOJ, Cuthbert NJ, Francis DL (1981). Model of opiate dependence in the guinea-pig isolated ileum. Br J Pharmacol.

[5] Ross GR, Gabra BH, Dewey WL, Akbarali HI (2008). Morphine tolerance in the mouse ileum and colon. J Pharmacol Exp Ther.

[6] Rezvani A, Huidobro-Toro JP, Hu J, Way EL (1983). A rapid and simple method for the quantitative determination of tolerance development to opiates in the guinea-pig ileum in vitro. J Pharmacol Exp Ther.

[7] Leedham JA, Kong JQ, Taylor DA, Johnson SM, Fleming WW (1992). Membrane potential in myenteric neurons associated with tolerance and dependence to morphine. J Pharmacol Exp Ther.

[8] Goldstein A, Schulz R (1973). Morphine-tolerant longitudinal muscle strip from guinea-pig ileum. Br J Pharmacol.

[9] Aleixandre MA, Colado MI, García de Jalón PD, Martín MI (1983). Influences on morphine action in the myenteric plexus from guinea pig of alpha 2 receptor stimulation. Arch Farmacol Toxicol.

[10] Mehr SE, Samini M, Namiranian K, Homayoun H, Gaskari SA, Dehpour AR (2003). Inhibition by immunophilin ligands of morphine-induced tolerance and dependence in guinea pig ileum. Eur J Pharmacol.

[11] Paton WD (1970). Drug dependence: pharmacological and physiological aspects. J R Coll Physicians Lond.

[12] Valeri P, Morrone LA, Romanelli L, Amico MC (1995). Acute withdrawal after bremazocine and the interaction between μ- and κ-opioid receptors in isolated gut tissues. Br J Pharmacol.

[13] Fichna J, Schicho R, Andrews CN, Bashashati M, Klompus M, Mckay DM (2009). Salvinorin A inhibits colonic transit and neurogenic ion transport in mice by activating κ-opioid and cannabinoid receptors. Neurogastroenterol Motil.

[14] Sałaga M, Polepally PR, Sobczak M, Grzywacz D, Kamysz W, Sibaev A (2014). Novel orally available salvinorin A analog PR-38 inhibits gastrointestinal motility and reduces abdominal pain in mouse models mimicking irritable bowel syndromes. J Pharmacol Exp Ther.

[15] Polepally PR, Huben K, Vardy E, Setola V, Mosier PD, Roth BL (2014). Michael acceptor approach to the design of new salvinorin A-based high affinity ligands for the kappa-opioid receptor. Eur J Med Chem.

[16] Cichewicz DL, Welch SP (2003). Modulation of oral morphine antinociceptive tolerance and naloxone-precipitated withdrawal signs by oral δ9-tetrahydrocannabinol. J Pharmacol Exp Ther.

[17] Basilico L, Parolaro D, Colleoni M, Costa B, Giagnoni G (1999). Cross-tolerance and convergent dependence between morphine and cannabimimetic agent WIN 55,212–2 in the guinea-pig ileum myenteric plexus. Eur J Pharmacol.

[18] Rodríguez JJ, Mackie K, Pickel VM (2001). Ultrastructural localization of the CB1 cannabinoid receptor in μ-opioid receptor patches of the rat caudate putamen nucleus. J Neurosci.

[19] Pickel VM, Chan J, Kash TL, Rodríguez JJ, Mackie K (2004). Compartment-specific localization of cannabinoid 1 (CB1) and μ-opioid receptors in rat nucleus accumbens. Neuroscience.

[20] Hojo M, Sudo Y, Ando Y, Minami K, Takada M, Matsubara T (2008). μ-Opioid receptor forms a functional heterodimer with cannabinoid CB1 receptor: Electrophysiological and fret assay analysis. J Pharmacol Sci.

[21] Remesic M, Hruby VJ, Porreca F, Lee YS (2017). Recent advances in the realm of allosteric modulators for opioid receptors for future therapeutics. ACS Chem Neurosci.

[22] Pertwee RG, Ross RA, Craib SJ, Thomas A (2002). (-)-Cannabidiol antagonizes cannabinoid receptor agonists and noradrenaline in the mouse vas deferens. Eur J Pharmacol.

[23] Rothman RB, Murphy DL, Xu H, Godin JA, Dersch CM, Partilla JS (2007). Salvinorin A: allosteric interactions at the μ-opioid receptor. J Pharmacol Exp Ther.

